# Demonstrating the Benefits of Antihypertensive Nighttime Dosing and Indapamide Usage in Hypertension Management

**DOI:** 10.1177/87551225231207275

**Published:** 2023-11-08

**Authors:** Jonathan S. Newsome

**Affiliations:** 1 Clinical Sciences Department, The Ben and Maytee Fisch College of Pharmacy, The University of Texas at Tyler, Tyler, TX, USA

**Keywords:** chronotherapy, nighttime, hypertension, indapamide, hydrochlorothiazide, pharmacist

## Abstract

**Background:** Uncontrolled hypertension, specifically nocturnal hypertension, increases the risk for significant clinical outcomes. Evidence on the use of nighttime antihypertensives is scant and conflicting. In addition, hydrochlorothiazide continues to be the primary thiazide used despite being the least potent. **Objective:** The primary purpose of this study was to evaluate instituting nighttime dosing to control hypertension and compare the short-term effectiveness of blood pressure control with indapamide versus hydrochlorothiazide. **Methods:** This was a retrospective, observational study. Participant inclusion criteria consisted of patients 18 years of age or older, a current diagnosis of hypertension, and hypertension that required medical therapy. The investigator documented whether a patient was taking at least one antihypertensive at night versus all morning medications, as well as the use of indapamide versus hydrochlorothiazide. The patient’s baseline and first follow-up blood pressure readings were documented. The primary outcome was to determine whether including nighttime dosing in antihypertensive regimens is more effective than all morning antihypertensive regimens. The secondary outcome was to determine whether indapamide was more effective than hydrochlorothiazide. **Results:** A total of 64 patients were included in the study. Twenty-eight patients were taking >1 nighttime antihypertensives versus 32 patients on all morning medications. Patients on at least one nighttime medication demonstrated greater systolic blood pressure reduction. There was no difference in blood pressure reduction between indapamide and hydrochlorothiazide. **Conclusion:** The study findings support the use of nighttime dosing to improve blood pressure management. The results on the effectiveness of indapamide versus hydrochlorothiazide conflict with previous research.

## Background

Hypertension is a significant health problem in the United States. Approximately 47% of US adults have defined hypertension (blood pressure ≥130/80 mm Hg) or are taking an antihypertensive. Only 24% of those adults are considered to have controlled hypertension.^1^ There are various barriers that affect the management of hypertension such as cultural beliefs, lack of awareness and education, insufficient access to culturally appropriate screening and treatment, and limited access to resources (eg, healthy foods and opportunities for physical activity).^2^ These barriers can result in life-threatening prognoses, including heart attack, stroke, and death. In 2021, hypertension directly or indirectly accounted for 691 095 deaths in the United States.^1^ Pharmacist intervention and management of hypertension is integral in the alleviation of life-threatening health outcomes and will contribute to an overall increased control of hypertension within underserved populations. Studies have shown that pharmacist intervention can significantly increase disease-related knowledge, blood pressure control, and medication adherence in patients with hypertension.^3^

One aspect of blood pressure control is addressing nocturnal hypertension. Nocturnal hypertension is defined as a nighttime blood pressure of ≥120/70 mm Hg.^4^ It is well documented that arterial blood pressure surges in the morning and during daytime activities, and decreases by 10% to 20% during sleep.^5^ However, in certain hypertensive patients, the blood pressure does not dip at night (non-dippers) or have higher nighttime readings than daytime readings (reverse dipping). Approximately 32% to 46% of adults with hypertension are considered non-dippers while 5% to 19% have reverse dipping. This increases the risk for significant clinical outcomes.^5^ Elevated nocturnal blood pressure has been associated with microalbuminuria in type 1 diabetics and left ventricular hypertrophy. Furthermore, nocturnal blood pressure may be more predictive of cardiovascular and all-cause mortality outcomes than systolic daytime readings.^6^

When addressing nocturnal hypertension, there is growing evidence that drug pharmacokinetics should be considered. One drug in particular is amlodipine. Thoonkuzhy and Rahman^7^ demonstrated that the time maximum plasma concentration was shorter, and the mean peak plasma concentration was greater when amlodipine was administered in the evening. In addition, amlodipine had a longer half-life when administered at night versus the day.^7^

Based on the literature, thiazide-like diuretics, such as indapamide, appear to be more effective than thiazide-type diuretics (hydrochlorothiazide [HCTZ]) in reducing the risk of major cardiovascular effects in people with hypertension. In the HYVET trial, indapamide (with the addition of perindopril, if necessary) versus placebo was studied to examine whether there was a significant difference in the reduction of fatal or nonfatal stroke incidence. Indapamide was associated with a statistically significant reduction in stroke, death from any cause, and heart failure compared with placebo.^8^ Indapamide has a longer half-life, confers more antihypertensive benefit, and important pleiotropic effects that may include micro- and macrovascular benefits in patients with diabetes while reducing microalbuminuria.^9^ Hydrochlorothiazide has traditionally been the thiazide of choice due to cost. However, with indapamide being a cost-effective medication as well, the literature suggests that indapamide could be preferred to HCTZ.

The purpose of this study is to (1) evaluate instituting nighttime dosing to control hypertension, specifically the use of amlodipine; (2) compare the short-term effectiveness of blood pressure control with indapamide versus hydrochlorothiazide; and (3) evaluate the effectiveness of a pharmacist-led hypertension program.

## Methods

This retrospective, observational study was conducted at a federally qualified health center in Tyler, TX. It was reviewed by the University of Texas at Tyler Institutional Review Board and approved on May 9, 2022. Participants of the study included patients who were referred for the pharmacist-led hypertension management program. In 2019, a protocol was approved and implemented by the administration of the clinic. This protocol allowed for patients to be referred to the pharmacist-led program if they have an elevated blood pressure without a hypertension diagnosis, are newly diagnosed, or have difficult to control hypertension for further management. The pharmacist would obtain the patient’s blood pressure, assess lifestyle modifications and pharmacotherapy, counsel on adherence and administration issues, provide recommendations to the provider, and follow-up with the patient. Participant inclusion criteria consisted of patients 18 years of age or older, a current diagnosis of hypertension, and hypertension that required medical therapy. Participant exclusion criteria consisted of patients younger than 18 years of age, patients without a diagnosis of hypertension, and patients with hypertension that did not require medical therapy. The charts of the patients meeting the inclusion criteria were reviewed by the primary investigator. The primary investigator documented the patient’s initial visit and the first follow-up visit after 1 month. The blood pressure at the initial visit served as the baseline reading, and the blood pressure at the follow-up visit was used to determine intervention impact. It was noted whether the patient was taking hydrochlorothiazide or indapamide. Furthermore, it was documented whether the patient was taking at least one nighttime antihypertensive versus all antihypertensives being taken in the morning. The primary outcome was to determine whether utilizing nighttime dosing in an antihypertensive regimen resulted in further blood pressure (BP) reduction compared to an all morning antihypertensive regimen. Furthermore, a subgroup of this outcome was to assess the effectiveness of amlodipine as the nighttime antihypertensive versus other antihypertensives (not including thiazides) as the nighttime agent. The secondary outcome was to determine whether indapamide was more effective at reducing systolic blood pressure (SBP) and diastolic blood pressure (DBP) compared with HCTZ. The tertiary outcome was to reinforce that pharmacist-led BP management programs significantly improve patient’s blood pressure readings. A 2-sample *t* test assuming equal variances was used to determine whether including nighttime dosing in the antihypertensive regimen resulted in a significant blood pressure reduction compared with patients only taking antihypertensives in the morning. In addition, a 2-sample *t* test assuming equal variances was used to compare the effectiveness of indapamide on SBP and DBP compared with hydrochlorothiazide. A paired *t* test was used to determine whether a significant reduction in SBP and DBP was observed between the initial visit and the follow-up visit.

## Results

A total of 64 patients were included in the study. The mean initial SBP and DBP for the enrolled patient was 148.8 and 90.3 mm Hg, respectively. At follow-up, the mean SBP and DBP was 131.3 and 83.6 mm Hg, respectively. Patients achieved an average SBP reduction of 17.5 mm Hg (*P* < 0.05) at first follow-up (Table 1). Furthermore, patients achieved an average DBP reduction of 6.7 mm Hg (*P* < 0.05) at first follow-up (Table 2). A total of 28 patients (46.7%) were taking at least one nighttime antihypertensive compared with 32 patients (53.3%) only taking morning antihypertensives. Patients taking an antihypertensive at nighttime observed a significant reduction in SBP (−24.4 mm Hg; *P* < 0.05) and DBP (−8.4 mm Hg; *P* < 0.05). In addition, the findings demonstrate that patients with a nighttime antihypertensive observed a more significant reduction in SBP compared with those without a nighttime agent. Patients receiving a nighttime antihypertensive achieved an SBP reduction of 24.4 mm Hg compared with a reduction of 11.4 mm Hg in patients only receiving morning antihypertensives (*P* < 0.05, Table 3). However, a statistically significant reduction was not observed with DBP (Table 4). Patients receiving a nighttime antihypertensive achieved a DBP reduction of 8.4 mm Hg compared with a 4.5 mm Hg reduction in patients only receiving morning antihypertensives (*P* = 0.15). In addition to examining nighttime dosing, the study assessed the difference in BP reduction when amlodipine was used as a nighttime antihypertensive versus another agent. Patients receiving amlodipine (n = 16; 57.1%) as the nighttime antihypertensive did not have a statistically significant (*P* = 0.75) reduction in SBP compared with when another agent (n = 12; 42.9%) was used. There was no significant difference observed in DBP. The study findings did not signify that indapamide therapy was more effective than HCTZ therapy or vice versa. A total of 11 patients (57.9%) were taking indapamide compared with 8 patients (42.1%) taking hydrochlorothiazide. Patients taking indapamide did achieve a statistically significant reduction in blood pressure (Table 5 & 6). When comparing the mean SBP and DBP reduction between indapamide and HCTZ, patients achieved a slightly greater reduction in DBP with indapamide (DBP: 10.5 mm Hg vs 7.9 mm Hg). However, this additional reduction was not statistically significant.

**Table 1.** Systolic Blood Pressure Reduction From Baseline to Initial Follow-up.

	Initial SBP	Follow-up SBP
Mean (mm Hg)	148.8	131.3
Observations	64	64
*P*(*T* ≤ *t*) 2-tail	<0.001	

Abbreviation: SBP, systolic blood pressure.

**Table 2.** Diastolic Blood Pressure Reduction From Baseline to Initial Follow-up.

	iDBP	fDBP
Mean (mm Hg)	90.3	83.6
Observations	64	64
*P*(*T* ≤ *t*) 2-tail	<0.001	

Abbreviations: fDBP, follow-up diastolic blood pressure; iDBP, initial diastolic blood pressure.

**Table 3.** Difference in Systolic Blood Pressure Reduction (pm Dosing vs am Dosing).

	Diff pm SBP	Diff am SBP
Mean (mm Hg)	24.4	11.4
Observations	28	32
*P*(*T* ≤ *t*) 2-tail	<0.05	

Abbreviation: SBP, systolic blood pressure.

**Table 4.** Difference in Diastolic Blood Pressure Reduction (pm Dosing vs am Dosing).

	Diff pm DBP	Diff am DBP
Mean (mm Hg)	8.4	4.5
Observations	28	32
*P*(*T* ≤ *t*) 2-tail	0.14	

Abbreviation: DBP, diastolic blood pressure.

**Table 5.** Difference in Diastolic Blood Pressure Reduction (HCTZ vs Indapamide).

	Diff HCTZ DBP	Diff Indap DBP
Mean (mm Hg)	7.9	10.5
Observations	8	11
*P*(*T* ≤ *t*) 2-tail	0.45	

Abbreviations: DBP, diastolic blood pressure; HCTZ, hydrochlorothiazide.

**Table 6.** Difference in Systolic Blood Pressure Reduction (HCTZ vs Indapamide).

	Diff HCTZ SBP	Diff Indap SBP
Mean (mm Hg)	24.6	24.5
Observations	8	11
*P*(*T* ≤ *t*) 2-tail	0.99	

Abbreviations: HCTZ, hydrochlorothiazide; SBP, systolic blood pressure.

## Discussion

Based on the findings, this study supports current literature that pharmacist involvement in blood pressure management can positively affect blood pressure readings.^10,11^ In this study, a pharmacist-led hypertension management program resulted in statistically significant improvement in SBP and DBP. This supports findings that a pharmacist-led intervention can result in BP reduction.^10^

Regarding nighttime dosing of antihypertensives, current literature has produced mixed results. Studies for nighttime dosing have either focused on BP lowering or cardiovascular outcomes. Hermida et al^12^ found that SBP was significantly lower in sleeping participants when administered nighttime antihypertensives versus morning antihypertensives. In addition, non-dipper status was significantly lower in participants receiving nighttime dosing. Furthermore, the proportion of patients classified as controlled was higher in patients taking at least one or more antihypertensives at bedtime. In contrast, the HARMONY (Hellenic-Anglo Research into Morning or Night Antihypertensive Drug Delivery) study findings found no significant differences in mean 24-hour ambulatory SBP and DBP or mean daytime or nighttime SBP and DBP based on morning or evening dosing.^13^ However, one limitation of the HARMONY study was participants either received all their medications in the morning or evening. This is a significant limitation because having an either-or method does not assess the benefit of using split dosing (one agent in the morning and one agent at night). In addition, the authors cited the composition of the cohort as the greatest limitation. The cohort for the study was relatively healthy, which is not the population who would benefit from nocturnal versus morning dosing. Non-dipping and nocturnal hypertension are more prevalent in higher risk patients.

The literature assessing improvement in cardiovascular outcomes with nighttime dosing is scant. Positive findings have been seen with the MAPEC trial and the HYGIA Chronotherapy Trial. The primary outcome of the MAPEC trial was total cardiovascular disease morbidity and mortality, which included death from all causes, myocardial infarction, angina pectoris, coronary revascularization, heart failure, acute arterial occlusion of the lower extremities, rupture of aortic aneurysms, thrombotic occlusion of the retinal artery, hemorrhagic stroke, ischemic stroke, and transient ischemic attack. Participants taking at least one or more medications at bedtime had significantly lower occurrences of the primary outcome compared with patients taking their medications upon awakening.^14^

The HYGIA Chronotherapy Trial showed that patients randomized to a bedtime regimen showed a significantly lower risk of the primary cardiovascular disease outcome.^15^ However, the HYGIA study was controversial due to concerns about how the study was conducted and reported. There was concern whether the study was truly randomized or were groups allocated. Study protocol discontinuation and patients’ loss to follow-up and withdrawals were not reported. Finally, insufficient information about medication adherence and adverse effects was disclosed.

The TIME study was recently published to investigate whether evening dosing improves major cardiovascular outcomes compared with morning dosing in patients with hypertension.^16^ This study concluded that there was no difference between morning dosing and evening dosing on cardiovascular outcomes. However, an observational cohort study found that nighttime SBP was the most informative indicator for all-cause and cardiovascular death risk.^17^

One possible limitation of using nighttime dosing is adherence. One analysis found that patients taking their medication in the morning were significantly more adherent than those taking their medication at night.^18^Another study found that adherence to amlodipine 5 mg once daily was higher in patients taking the medication in the morning compared with night.^19^ A method to improve adherence is to schedule follow-up communication with the patient to ensure adherence. Smith et al had student pharmacist give each participant a personal medication record with instructions regarding the once-daily antihypertensive medication assigned to morning or evening dosing. Participants were then contacted 3 to 6 weeks after the visit by the student to assess adherence using the Morisky Medication Scale. The study findings showed that 91% of patients on the morning medication were adherent compared with 95% of patients on the evening medication. This study demonstrated that a small, brief follow-up encounter with a health care professional such as a pharmacist can have a significant impact on medication adherence.^20^

The current study’s findings regarding indapamide effectiveness versus HCTZ conflict with previous literature. Although patients in this study achieved a slightly greater reduction in DBP with indapamide versus HCTZ, this reduction was not statistically significant. In addition, there was no difference in SBP reduction. In contrast, previous studies have demonstrated that indapamide produced a greater reduction in SBP than HCTZ.^21^One possible explanation for this discrepancy in results is the sample size. Only 8 and 11 participants were taking HCTZ and indapamide in this study, respectively.

This study does have multiple limitations that should be addressed. As stated previously, sample size is a limitation. The study only included 64 patients. In addition, extrapolation of results to the general population will be difficult due to the lack of reporting on patient characteristics such as gender and race. Finally, the duration of the study (follow-up was assessed after 1 month) does not provide a long-term perspective on the effects of therapy.

## Conclusion

The study results further strengthen previous evidence that pharmacist-led BP management programs are effective for improving BP levels. The study implies that emulating circadian rhythm (having at least one nighttime medication as part of the BP regimen) results in greater SBP reduction than only taking antihypertensives in the morning. Larger trials should continue to be conducted to reaffirm these results and provide additional insight into how emulating circadian rhythm can affect cardiovascular outcomes. The study results conflict with current literature regarding indapamide effectiveness versus HCTZ. However, limitations in the study could have skewed these results.
